# Extracorporeal Membrane Oxygenation in Pediatric Severe Aplastic Anemia: A Case Report Highlighting Risk-Benefit and Ethical Considerations

**DOI:** 10.7759/cureus.105764

**Published:** 2026-03-24

**Authors:** Abigail S Abraham, Merin M Mathew, Lyndsey Thomas, John Abraham, Madhura Butala

**Affiliations:** 1 College of Osteopathic Medicine, Lake Erie College of Osteopathic Medicine, Bradenton, USA; 2 Research, Lake Erie College of Osteopathic Medicine, Bradenton, USA; 3 Molecular Cellular Developmental Biology, University of California Santa Barbara, Santa Barbara, USA; 4 Pediatrics, Ascension St. Vincents, Jacksonville, USA

**Keywords:** bone marrow failure, case report, extracorporeal membrane oxygenation, hematology, pediatrics, risk–benefit analysis, septic shock, severe aplastic anemia

## Abstract

Severe aplastic anemia (SAA) is a rare pediatric bone marrow failure disorder marked by profound pancytopenia and high risk of infection, bleeding, and organ dysfunction. Despite advances in definitive therapy, children with SAA remain vulnerable to rapid deterioration during neutropenic sepsis. The role of extracorporeal membrane oxygenation (ECMO) in this population is poorly defined due to substantial hemorrhagic, thrombotic, and infection risks, thus necessitating careful risk-benefit assessment. A four-year-old child presented with febrile neutropenia and profound pancytopenia and was diagnosed with idiopathic severe aplastic anemia based on bone marrow biopsy demonstrating marked hypocellularity. Her course was complicated by persistent marrow failure and recurrent infections despite supportive care. Four months later, she developed refractory septic shock requiring ECMO, which was complicated by severe bleeding, disseminated intravascular coagulation, venous thrombosis, and progressive multi-organ failure, resulting in death. This case highlights the narrow therapeutic window of ECMO in pediatric SAA, in which profound cytopenias and immunosuppression markedly increase morbidity. While ECMO may provide temporary cardiopulmonary stabilization, its benefit depends on the likelihood of hematologic recovery and a clear pathway to definitive therapy. The use of ECMO in pediatric severe aplastic anemia, therefore, requires cautious patient selection, early multidisciplinary collaboration, and proactive goals-of-care discussions to ensure alignment with prognosis and family-centered values.

## Introduction

Severe aplastic anemia (SAA) is a rare, life-threatening bone marrow failure disorder characterized by profound pancytopenia and a high risk of infection, bleeding, and organ dysfunction [1-3]. Despite advances in immunosuppressive therapy and hematopoietic stem cell transplantation, pediatric patients remain vulnerable to rapid clinical deterioration, particularly during episodes of neutropenic sepsis [4-6]. When deterioration progresses to refractory circulatory failure, extracorporeal membrane oxygenation (ECMO) may be considered if conventional therapies fail. However, the use of ECMO in pediatric SAA is poorly studied and may be complicated by thrombocytopenia, coagulopathy, and immunosuppression [7-9]. 

We describe the case of a four-year-old child with idiopathic severe aplastic anemia who developed refractory septic shock requiring ECMO, highlighting the clinical, ethical, and risk-benefit considerations of advanced life support in pediatric bone marrow failure syndromes. 

## Case presentation

A four-year-old nonverbal female patient with autism and no prior hematologic history presented with five days of fever and cough, a febrile seizure, periorbital ecchymosis, and oral mucosal bleeding. Examination revealed fever, facial ecchymosis, and oral bleeding, with initially clear lung auscultation. Initial laboratory evaluation revealed profound pancytopenia, including severe anemia (hemoglobin 3.4 g/dL), marked thrombocytopenia (platelets 5 K/µL), and near-absent neutrophils (absolute neutrophil count 0.06 K/µL), consistent with febrile neutropenia. Coagulation studies demonstrated significant coagulopathy with prolonged prothrombin time (PT) and elevated international normalised ratio (INR), with a normal partial thromboplastin time. Additional metabolic abnormalities were present (Tables 1-2).

**Table 1:** Complete blood count (CBC) and coagulation studies at initial presentation *Abnormal values are flagged according to institutional laboratory reference ranges. "Critical" denotes critically abnormal values requiring urgent clinical attention. MCV: mean corpuscular volume; MCH: mean corpuscular hemoglobin; MCHC: mean corpuscular hemoglobin concentration; MPV: mean platelet volume; PT: prothrombin time; INR: international normalized ratio; PTT: partial thromboplastin time

Laboratory Category	Test	Value	Reference Range (Pediatric)
Hematologic	White Blood Cell Count	2.10 K/mcL *Low	5.0–14.5 K/mcL
	Red Blood Cell Count	1.27 million/mcL *Low	3.9–5.3 million/mcL
	Hemoglobin	3.4 g/dL *Critical	11.5–14.5 g/dL
	Hematocrit	9.9 % *Critical	33–43 %
	MCV	78.6 fL *Low	80–94 fL
	MCH	26.7 pg	24–30 pg
	MCHC	34.0 g/dL	32–36 g/dL
	Red Cell Distribution Width	15.5 % *High	11.5–14.5 %
	Platelet Count	5 K/mcL *Critical	150–450 K/mcL
	MPV	9.9 fL	7.5–11.5 fL
	Neutrophils – Absolute Manual	0.06 K/mcL *Critical	1.5–8.0 K/mcL
	Manual Segmented Neutrophil %	3 % *Low	30–55 %
	Manual Lymphocytes %	97 % *High	30–60 %
	Nucleated Red Blood Cells	0	0
	RBC Morphology	*Abnormal	Normal
	Anisocytosis	Slight	None
	Poikilocytosis	Slight	None
	Microcytes	Few	None
	Macrocytes	Few	None
	Ovalocytes	Few	None
	Platelet Sufficiency	*Markedly decreased	Adequate
Coagulation	PT	23.8 sec *High	11–14 second
	INR	2.2 *High	0.9–1.2
	PTT	26.0 sec	25–35 second

**Table 2:** Comprehensive metabolic panel (CMP) at initial presentation *Abnormal values are flagged according to institutional laboratory reference ranges. A/G ratio: albumin/globulin ratio; AST: aspartate aminotransferase; ALT: alanine aminotransferase

Test	Value	Reference Range (Pediatric)
Sodium	129 mmol/L *Low	135–145 mmol/L
Potassium	3.4 mmol/L *Low	3.5–5.0 mmol/L
Chloride	95 mmol/L *Low	98–107 mmol/L
Carbon Dioxide (Bicarbonate)	20 mmol/L *Low	22–28 mmol/L
Glucose	114 mg/dL *High	70–110 mg/dL
Blood Urea Nitrogen	19 mg/dL	7–20 mg/dL
Creatinine	0.6 mg/dL	0.3–0.7 mg/dL
Anion Gap	14 mmol/L	8–16 mmol/L
Osmolality (Calculated)	274 mOsm/kg *Low	275–295 mOsm/kg
Total Protein	5.9 g/dL	6.0–8.0 g/dL
Albumin	3.2 g/dL *Low	3.5–5.0 g/dL
A/G Ratio	1.2	1.0–2.5
Calcium	8.1 mg/dL *Low	8.5–10.5 mg/dL
Alkaline Phosphatase	92 intUnit/L *Low	150–420 intUnit/L
AST	31 intUnit/L	10–40 intUnit/L
ALT	12 intUnit/L	7–56 intUnit/L
Total Bilirubin	0.6 mg/dL	0.2–1.2 mg/dL
Lactic Acid	1.7 mmol/L	0.5–2.2 mmol/L

The patient was treated empirically for febrile neutropenia with intravenous fluids and cefepime for presumed pneumonia and otitis media. Following initial stabilization, the patient received a packed red blood cell transfusion and a platelet transfusion with improvement in hemoglobin and platelet count. She remained hemodynamically stable, without active bleeding or respiratory compromise. After clinical improvement and shared decision-making with the family, she was discharged with close outpatient hematology follow-up and strict return precautions pending bone marrow biopsy and further evaluation.

She re-presented two days later with clinical deterioration requiring pediatric intensive care admission. Formal illness severity scores were not available for retrospective calculation. Bone marrow biopsy revealed marked hypocellularity (~5%) with severe trilineage hypoplasia and no blasts or dysplasia, confirming very severe aplastic anemia under modified Camitta criteria. Cytogenetic analysis was performed to differentiate severe aplastic anemia from hypocellular myelodysplastic syndromes. Paroxysmal nocturnal hemoglobinuria (PNH) clone testing was not available for review. Extensive infectious and inherited marrow failure evaluation, including Fanconi anemia testing, was negative. Genetic testing identified variants of uncertain significance not felt to be contributory.

Her course was complicated by prolonged hospitalization and recurrent admissions for febrile neutropenia and pneumonia requiring extended intravenous antimicrobial therapy. She developed poor oral intake with micronutrient deficiencies necessitating nasogastric feeding. She continued to experience persistent severe pancytopenia, electrolyte abnormalities, and recurrent infections. She received broad-spectrum intravenous antimicrobial therapy for febrile neutropenia, with escalation based on clinical deterioration and concern for resistant organisms. Empiric antifungal coverage was initiated during episodes of persistent febrile neutropenia in accordance with institutional guidelines. During these hospitalizations, transfusion support was provided. Packed red blood cells were transfused for hemoglobin <7 g/dL or symptomatic anemia. Platelet transfusions were administered for platelet counts <10 ×10⁹/L or <20 ×10⁹/L in the setting of fever or active bleeding. Fresh frozen plasma and cryoprecipitate were administered during episodes of coagulopathy with active bleeding.

The patient underwent evaluation for hematopoietic stem cell transplantation after initial diagnosis. HLA typing for a sibling donor was underway. However, before the transplant planning could be completed, four months after initial diagnosis, she was rehospitalized with neutropenic fever and septic shock that progressed to circulatory collapse requiring venoarterial (VA) ECMO. Her course was complicated by neutropenic sepsis, severe thrombocytopenia, hematemesis, suspected disseminated intravascular coagulation, encephalopathy, capillary leak syndrome, and left cephalic vein thrombosis. Disseminated intravascular coagulation (DIC) was diagnosed based on thrombocytopenia, prolonged PT/INR, hypofibrinogenemia, elevated D-dimer levels, and clinical bleeding.

After one week of ECMO support, she developed ventricular tachycardia and multiorgan failure despite maximal therapy. She eventually died from complications of idiopathic severe aplastic anemia, septic shock, and ECMO-associated coagulopathy.

## Discussion

Epidemiology and pathology of aplastic anemia 

Aplastic anemia is a bone marrow failure disorder characterized by pancytopenia due to impaired hematopoiesis, with an incidence of approximately 2-2.5 pediatric cases per million annually in the United States and a bimodal age distribution, whereas the incidence in Asia ranges from four to seven cases per million annually [2,4]. This geographic variability is also observed in pediatric populations, in whom aplastic anemia remains rare but represents a significant cause of acquired bone marrow failure. Most cases are idiopathic, though inherited and acquired etiologies must be considered, particularly in pediatric patients [3]. Inherited bone marrow failure syndromes include Fanconi anemia, dyskeratosis congenita, and CTLA-4 deficiency, while acquired causes include toxic exposures, viral infections, nutritional deficiencies, and immune-mediated marrow suppression, as shown in Table 3 [1,3,10-11].

**Table 3:** Differential diagnosis and evaluation of aplastic anemia FISH: fluorescence in situ hybridization; GATA2: GATA-binding protein 2; CTLA-4: cytotoxic T-lymphocyte-associated protein 4; HIV: human immunodeficiency virus; PCR: polymerase chain reaction; GPI: glycosylphosphatidylinositol References: [1,3,10-11]

Category	Diagnosis	Diagnostic Evaluation
Inherited	Fanconi anemia	Chromosomal breakage analysis with diepoxybutane (DEB)
	Dyskeratosis congenita	Telomere length assessment (flow-FISH 6-cell panel)
	GATA2 deficiency	Germline genetic testing
	CTLA-4 deficiency	Germline genetic testing
Acquired	Chemotherapy or radiation exposure	Treatment history review
	Drug-induced marrow suppression	Medication review
	Nutritional deficiencies	Serum micronutrient testing
	Toxin exposure	Environmental exposure history
	Viral hepatitis	Hepatitis serologic testing
	Human immunodeficiency virus	HIV antigen/antibody testing
	Epstein–Barr virus, cytomegalovirus	PCR testing
	Parvovirus B19	PCR testing
	Thymoma	Chest imaging
	Paroxysmal nocturnal hemoglobinuria	High-sensitivity flow cytometry for GPI-anchored proteins
	Eosinophilic fasciitis	Clinical diagnosis ± biopsy

Immune-mediated aplastic anemia results from cytotoxic T-cell-driven destruction of hematopoietic stem cells and cytokine-mediated inhibition of progenitor cell self-renewal, leading to the characteristic finding of hypocellular, fatty bone marrow. Key inflammatory cytokines, particularly interferon-γ and tumor necrosis factor-α, play central roles in this process by inducing stem cell apoptosis, suppressing progenitor proliferation, and impairing the bone marrow microenvironment [4]. 

Clinical features and diagnosis 

Aplastic anemia presents with symptoms related to cytopenias, including fatigue, recurrent infections, mucocutaneous bleeding, petechiae, and bruising. Disease severity is classified using the modified Camitta criteria, with severe aplastic anemia defined by bone marrow cellularity <25% and at least two of the following: absolute neutrophil count <0.5 × 10⁹/L, platelet count <20 × 10⁹/L, or absolute reticulocyte count <60 × 10⁹/L; very severe disease is distinguished by an absolute neutrophil count <0.2 × 10⁹/L [2,4]. Diagnosis is established by bone marrow biopsy demonstrating marked hypocellularity without malignant infiltration. Immunohistochemical and reticulin staining aid in differentiating aplastic anemia from myelodysplastic syndromes [3]. A systematic evaluation, as shown in Table 3, is required to exclude inherited and acquired causes of pediatric bone marrow failure [1,3,10-11]. 

Treatment 

Once associated with high mortality, aplastic anemia has become largely treatable with advances in supportive care and definitive therapy with overall survival rates of 88-93% at five years [3,6] First-line treatment for children with severe aplastic anemia is matched sibling/related donor hematopoietic stem cell transplantation, with immunosuppressive therapy recommended when a matched sibling/related donor is unavailable [4-6,12]. Although overall survival is comparable between transplantation and immunosuppression, transplantation offers superior failure-free and event-free survival [4]. Bone marrow is the preferred graft source due to lower graft-versus-host disease risk and improved survival, and fresh grafts are favored over cryopreserved products [3,4,12].

Standard immunosuppressive therapy consists of horse-derived antithymocyte globulin combined with cyclosporine and corticosteroids used adjunctively to mitigate infusion-related reactions [4,5,12]. Thrombopoietin receptor agonists have demonstrated improved hematologic responses when added to standard immunosuppressive therapy in adults; however, optimal timing and long-term outcomes in children remain under investigation. Granulocyte colony-stimulating factor is no longer routinely recommended. Androgens such as danazol retain a role in select inherited marrow failure syndromes, particularly in telomere biology disorders [3,4,6,12]. 

ECMO use in pediatric aplastic anemia

ECMO provides advanced cardiopulmonary support in refractory cardiac or respiratory failure but poses unique challenges in patients with severe aplastic anemia due to profound pancytopenia, impaired hemostasis, and heightened infection risk [8]. In this case, ECMO was initiated for refractory septic shock in the setting of severe neutropenia and thrombocytopenia, refractory to conventional therapies. Refractory septic shock is defined as persistent tissue hypoperfusion requiring administration of ≥2 vasopressors with evidence of ongoing tissue hypoperfusion despite adequate fluid resuscitation and appropriate antimicrobial therapy [13]. However, complications arose, including major bleeding, suspected disseminated intravascular coagulation, and venous thrombosis, illustrating the narrow therapeutic window of ECMO in bone marrow failure syndromes [9,14,15].

The potential risks and benefits of ECMO in aplastic anemia 

The decision to initiate ECMO in patients with aplastic anemia requires careful consideration of its potential benefits and substantial risks. ECMO may provide temporary cardiopulmonary stabilization, allowing time for antimicrobial therapy, immunosuppressive modulation, or progression toward definitive treatment such as hematopoietic stem cell transplantation [7,8]. In select cases with reversible organ dysfunction and a clear pathway to hematologic recovery, ECMO may function as a bridge to survival [7].

However, these potential benefits must be weighed against significant risks. Severe thrombocytopenia, systemic anticoagulation, and consumptive coagulopathy increase the risk of life-threatening hemorrhage and thrombosis, while profound immunosuppression heightens susceptibility to overwhelming infection [8,9]. Severe coagulopathy or a contraindication to anticoagulation, including profound thrombocytopenia, is considered a relative contraindication to ECMO, as each 10 × 10⁹/L decrease in platelet count has been associated with an approximately 3.7% increase in bleeding risk [16]. In the general ECMO population, platelet counts are typically maintained at 80-100 × 10⁹/L. However, in patients with profound thrombocytopenia, current recommendations suggest maintaining platelet counts ≥40 × 10⁹/L during ECMO support [17]. Registry data from the Extracorporeal Life Support Organization (ELSO) indicate that immunocompromised patients have lower survival following ECMO compared with the general pediatric ECMO population. Reported survival among pediatric hematology-oncology patients supported with ECMO ranges from 52-64%, with survival to hospital discharge substantially lower at 36-42%, underscoring the distinction between short-term physiologic support and meaningful recovery [18,19]. 

Multidisciplinary care and goals-of-care considerations

In this case, persistent marrow failure and ongoing immunosuppression precluded hematologic recovery and contributed to severe infection and multiorgan failure. This outcome underscores the importance of early multidisciplinary collaboration among pediatric critical care, hematology, infectious disease, transfusion medicine, ethics, and palliative care teams when considering ECMO in children with aplastic anemia [7,14,20]. 

Early and ongoing goals-of-care discussions are essential to align ECMO initiation and continuation with realistic prognostic expectations, the likelihood of marrow recovery or transplantation, and patient- and family-centered values. Ideally, these discussions should occur prior to ECMO initiation whenever feasible, particularly in patients with severe underlying disease and uncertain potential for recovery. Ethical decision-making in this context often involves balancing the principles of proportionality and the pediatric best-interest standard while considering whether advanced life-sustaining therapies offer a meaningful chance of recovery. In some circumstances, ECMO may be approached as a time-limited trial of support, allowing clinicians and families to reassess the patient’s clinical trajectory, response to therapy, and evolving treatment options. Therefore, in the absence of a clear pathway to hematologic recovery, careful deliberation is required to ensure that ECMO functions as a meaningful bridge to recovery [14,15,18,20]. 

## Conclusions

Severe aplastic anemia is a rare pediatric bone marrow failure disorder in which ECMO may offer temporary cardiopulmonary support during refractory critical illness. This case highlights the potential benefits and limitations of ECMO and emphasizes the need for careful patient selection, early multidisciplinary collaboration, and proactive goals-of-care discussions when advanced life support is considered.
